# Effect of Nano-Silica and Sorbitol on the Properties of Chitosan-Based Composite Films

**DOI:** 10.3390/polym15194015

**Published:** 2023-10-07

**Authors:** Wei Zhang, Wentao Zhou, Zisen Zhang, Di Zhang, Zhengzheng Guo, Penggang Ren, Fei Liu

**Affiliations:** 1 Faculty of Printing, Packaging Engineering and Digital Media Technology, Xi’an University of Technology, Xi’an 710048, China; zhangwei@xaut.edu.cn (W.Z.); xutwentao@163.com (W.Z.); 18829029028@163.com (Z.G.); 2 School of Mechanical and Precision Instrument Engineering, Xi’an University of Technology, Xi’an 710048, China15797716937@163.com (D.Z.); 3 School of Materials Science and Engineering, Xi’an Polytechnic University, Xi’an 710048, China

**Keywords:** biomass chitosan, sorbitol, nano-silica, tensile strength, elongation at break, thermal stability

## Abstract

Chitosan and its derivatives are widely used in food packaging, pharmaceutical, biotechnology, medical, textile, paper, agriculture, and environmental industries. However, the flexibility of chitosan films is extremely poor, which limits its relevant applications to a large extent. In this paper, chitosan/sorbitol/nano-silica (CS/sorbitol/SiO_2_) composite films were prepared by the casting film method using chitosan, sorbitol, Tween-80 and nano-SiO_2_ as raw materials. The structure of the films was characterized by infrared spectroscopy, electron scanning microscopy, and X-ray diffraction analysis. The effects of sorbitol and nano-silica dosage on the mechanical properties, thermal properties and water vapor barrier properties of the composite film were investigated. The results show that with the gradual increase in sorbitol (≤75 wt %), the elongation at the break of chitosan/sorbitol films significantly increased. When the addition of sorbitol was 75 wt %, the elongation at break of the chitosan/sorbitol composite film was 13 times higher than that of the chitosan film. Moreover, nano-SiO_2_ can further improve the mechanical properties and thermal stability of the chitosan/sorbitol composite films. When the amount of nano-silica was 4.5 wt %, the composite film became more flexible, with a maximum elongation of 90.8% (which is 14 times that of chitosan film), and its toughness increased to 10.52 MJm^−3^ (which is 6 times that of chitosan film). This study balances the tensile strength and elongation at break of the composite films by adding a plasticizer and nano-filler, providing a reference for the preparation of chitosan composites or their blending with other polymers, and has practical guiding significance for the industrial production of biomass plastics.

## 1. Introduction

Most of the packaging materials made of petroleum-based polymers cause serious environmental pollution owing to their non biodegradable properties [1]. Environmentally friendly bio-polymers are increasingly favored due to their biocompatibility, biodegradability, non-toxicity, antibacterial activity, good transparency, ease of processing, and reusability. These materials are usually prepared from biopolymers such as proteins, lipids, polysaccharides and resins [2]. Furthermore, since no microplastics are produced during the degradation process, these biopolymers pose no threat to human health [3,4].

Chitosan (CS) is a natural cationic polysaccharide copolymer obtained from chitin, and is one of the most abundant polysaccharides on earth [5]. Chitosan has many advantages, such as its biocompatibility and biodegradability, as well as being non-toxic, which means that it can promote wound-healing and has a certain antibacterial ability [6]. In addition, chitosan also has a good film-forming capacity, and is widely used in food packaging, pharmaceutical, and agriculture industries, as well as other fields [7]. Due to the presence of amino groups, chitosan can dissolve in a dilute acid solution with a pH below 6.5 [8]. Therefore, acids such as acetate or lactate are often used to dissolve chitosan in the preparation of chitosan complexes [7]. Moreover, amino groups in chitosan also provide chitosan with functional properties: Electronegative amino groups absorb protons and have a positive charge, giving chitosan a variety of chemical, physical and biological properties, which is one of the reasons for its wide application [9,10].

Despite the many potential applications of chitosan films, studies have reported that unmodified pure films are fragile and brittle, which largely limits their application [2]. Therefore, it is necessary to improve the physical and mechanical properties of chitosan film. The key to preparing composite films with excellent flexibility is to destroy the intermolecular forces and improve the fluidity of the polymer molecular chains to weaken the brittleness of the material [11,12]. Plasticizers play an important role in modifying the physical and mechanical properties of composite film [13]. Adding a plasticizer to the film preparation process can reduce the friction between the polymer chains. The plasticizer molecules between the polymer chains break the hydrogen bond and separate the chains, which not only increases the flexibility of the film, but also improves the water vapor transmittance performance [14,15,16]. Consequently, many studies have mixed different kinds of plasticizers into composite films [17,18,19,20]. In order to address the shortcomings of chitosan films, Thakhiew et al. [17] prepared different chitosan-based composite films via three drying methods combined with four glycerol concentrations (0%, 25%, 75% and 125% *w*/*w* of chitosan). It was found that the drying methods and plasticizer concentration significantly affected the drying time, tensile strength, elongation at break and glass transition temperature of the films. Leceta et al. [18] prepared chitosan-based films plasticized with glycerol through casting. The films exhibited excellent barrier properties against water vapor and oxygen, and provided environmentally friendly materials for packaging applications. Mei et al. [19] investigated the effects of hydrophilic glycerol and hydrophobic perilla oil on the physicochemical, mechanical, optical and structural properties of starch–chitosan films. The results of Dayarian et al. [20] show that the chitosan and carboxymethyl chitosan films have promising applications in food packaging in terms of their water vapor and oxygen barrier properties. The combination of polyethylene glycol 400 and diethyl phthalate can improve the mechanical properties of the pectin composite films [13]. The plasticizers of 1-butyl-3-methylimidazolium chloride (BMIMCl) and lithium chloride (LiCl) increased the fluidity of the cellulose molecular chain, and caused the breaking elongation of the cellulose composite film to significantly increase [11]. Polyols have become the most-used plasticizer by virtue of their excellent plasticizing efficiency, large availability and reduced exudation, with an outstanding plasticizing effect on polysaccharide-based films.

In addition, nano-materials (nano-titanium dioxide, nano-silicon dioxide, nano-cellulose, etc.) can be added to the polymer matrix to form nano-composite materials with enhanced mechanical properties, which is also one of the common methods to improve the mechanical properties of composite films [6,21]. Nano-silica (SiO_2_) has good surface stability and a large specific surface area in acidic media, ideal thermal stability, resistance to microbial attack and a low cost. This can be combined with polymer compounds through chemical bonds or physical interaction, endowing polymer materials with special properties and leading to high-quality nanomaterials [22,23]. However, due to the existence of a large number of active silicon hydroxyl groups on the surface of nano-SiO_2_ particles, the “agglomeration phenomenon” of nanomaterials easily occurs, meaning that it is unable to maximize phase enhancement [24,25]. Therefore, nano-SiO_2_ needs to be functionalized with organic molecules and physically modified by surface activity treatment to be well-distributed in the polymer. By adding nano-silica to a poly(butylene adipate-co-terephthalate)/thermoplastic hydroxypropyl film with a high hydroxypropyl starch content, the horizontal tensile strength and vertical tensile strength of the composite film were increased to varying degrees [26]. In the study of Zhu et al. [27], nanosilica enhanced the mechanical properties of starch-based films and increased the tensile strength by 54%.

In this work, chitosan/sorbitol/SiO_2_ composite films with different sorbitol and nano-SiO_2_ mass ratios were successfully prepared using the casting film method. The effects of sorbitol and nano-SiO_2_ additions on the structure, surface morphology, mechanical properties, thermal properties and barrier properties of chitosan/sorbitol/SiO_2_ composite films were systematically examined.

## 2. Materials and Methods

### 2.1. Materials

Chitosan (CS, average molecular weight of 7 × 10^5^~8 × 10^5^ g/mol and deacetylated of 90%) was purchased from Hai Lan Ji Technology Development Co., Ltd. (Hai Lan Ji Technology Development Co., Ltd., Shanghai, China). Sorbitol (analytical reagent) and Tween-80 were from Tianjin Tianli Chemical Reagent Co., Ltd. (Tianli Chemical Reagent Co., Ltd., Tianjin, China). Glacial acetic acid was provided by Fuyu Fine Chemical Co., Ltd. (Fuyu Fine Chemical Co., Ltd., Tianjin, China). Nanosilica (with an average particle size of 100 nm) was purchased from Shanghai Kaiyin Chemical Co., Ltd. (Kaiyin Chemical Co., Ltd., Shanghai, China).

#### 2.1.1. Preparation of CS/Sorbitol Composite Films

**Figure 1:**
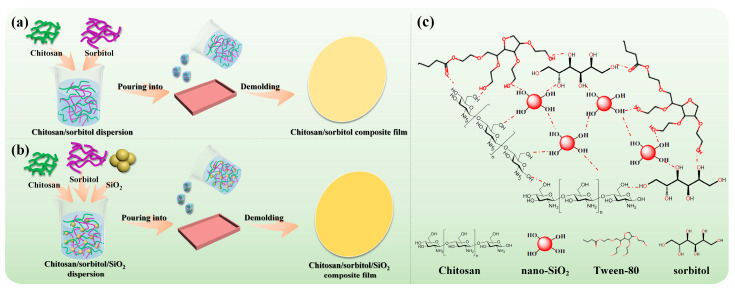
Preparation process diagram of the (**a**) chitosan/sorbitol composite films, and (**b**) chitosan/sorbitol/SiO_2_ composite films. (**c**) The structural scheme of the interaction between chitosan, sorbitol, Tween-80 and nano-SiO_2_.

Figure 1a shows the preparation process of the chitosan/sorbitol composite films. 2% (*w*/*v*) chitosan powders were dissolved in 50 mL of 2% (*v*/*v*) acetic acid solution. A different amount (30%, 45%, 60%, and 75% *w*/*w*) of sorbitol was added in 2% chitosan solution and stirred at 70 °C for 30 min. After naturally cooling to room temperature, the mixed solution was poured into a PTFE mold (80 mm × 80 mm), then placed in a constant temperature and humidity box with a temperature of 50 °C and humidity of 50% for 24 h. CS_30_, CS_45_, CS_60_ and CS_75_ represent chitosan with the addition of 30%, 45%, 60%, and 75% (*w*/*w*) sorbitol, respectively.

#### 2.1.2. Preparation of CS/Sorbitol/SiO_2_ Composite Films

The preparation process of the chitosan/sorbitol/nanosilica (CS/sorbitol/SiO_2_) composite films is described in Figure 1b. CS_60_ was selected for further research with 2.5%, 4.5%, and 6.5% (*w*/*w*) of nano-SiO_2_ powders added to three different beakers containing 49 mL of deionized water (0.5 g Tween-80 as dispersant), respectively, and stirred with a magnetic stirrer at room temperature for 45 min. For the concentration of nano-SiO_2_, refer to the work of Marangoni et al. [28]. These were dispersed with ultrasonic (ultrasonic power 120 W, ultrasonic time 30 min) and magnetic stirring at room temperature for 45 min. A total of 1 mL of acetic acid solution, 2% chitosan and 60% sorbitol were added to the dispersed nano-SiO_2_ powdersTween solution and stirred at 70 °C for another 30 min to obtain CS/sorbitol/SiO_2_ composite films, named CS_60_/SiO_2_-2.5, CS_60_/SiO_2_-4.5, and CS_60_/SiO_2_-6.5, respectively. Figure 1c shows the structural scheme, highlighting the interaction between chitosan, Tween-80 and SiO_2_.

### 2.2. Characterization

#### 2.2.1. Fourier-Transform Infrared (FTIR) Spectroscopy

The samples were scanned within the wavelength range of 4000–400 cm^−1^ using Nicolet iS50 Fourier transform infrared spectrometer, with a resolution of 2 cm^−1^ and a scanning frequency of 32 times. The data were analyzed using Origin v2021 software (Origin Lab Corporation, Northampton, MA, USA).

#### 2.2.2. X-ray Diffraction (XRD)

The crystalline structure of the composite films was analyzed using a DX-2700BH X-ray diffractometer. The tube voltage was 40 kV, the tube current was 30 mA, and the scanning rate was 10°/min. All the films were performed within the 2θ range of 5~40°, with a step size of 0.02°.

#### 2.2.3. Scanning Electron Microscopy (SEM)

A scanning electron microscope (SU8000, JEOLCompact, Tokyo, Japan) was used to observe the morphology of all composite films. The films were brittlefractured by freezing with liquid nitrogen and sputtered with a gold layer. The images were observed at an accelerating voltage of 10.0 kV and current of PC40.

#### 2.2.4. Mechanical Properties of the Composite Films

Mechanical properties were measured with a C610 intelligent electronic tensile testing machine according to ASTM D638 [29]. The composite films were cut into strips (8 cm × 1 cm) with a razor blade. The thickness of the sample was measured using a C640 thickness gauge at three different positions. Then, samples were tightened onto the fixture of the electronic tensile testing machine, with the initial fixture spacing and stretching speed set to 50 mm and 50 mm/min, respectively. The tensile strength (TS) and elongation at break (EAB) were calculated using Equation (1) and Equation (2), respectively:

TS (MPa) = F_max_/S (1)

where F_max_ is the maximum load, and S is the cross-sectional area of the film.

EAB (%) = (L − L_o_)/L_o_ × 100 (2)

where L is the length at which the film breaks, and L_o_ is the initial length of the film sample.

#### 2.2.5. Thermogravimetric Analysis (TG)

Thermogravimetric analysis of sample films was performed by a thermogravimetric analyzer (TG209F3 Tarsus, Selb, Germany) ranging from 30 °C to 500 °C with a heating rate of 10 °C/min. The test was conducted under a nitrogen atmosphere with a flow rate of 30 mL/min. Each sample was tested with a mass of approximately 5–10 mg.

#### 2.2.6. Dynamic Mechanical Analysis (DMA)

The DMA 242E dynamic thermo-mechanical analyzer (DMA) of NETZSCH was used for the test. The experiment was conducted at a temperature range of 30~200 °C and a heating rate of 5 °C/min. The gas flow rate was 20 mL/min in nitrogen atmosphere.

#### 2.2.7. Differential Scanning Calorimetry (DSC)

Differential scanning calorimetry (DSC) measurements were performed by a DSC200F3 (NETZSCH, Selb, Germany). The first heating increased from 0 °C to 200 °C and was kept at 200 °C for 3 min to eliminate the thermal history of the material. Then, at a cooling rate of 10 °C/min, the temperature dropped to 30 °C. The second heating process increased to 200 °C with a heating rate of 10 °C/min. The thermal properties of the composite film during the second temperature rise were analyzed.

#### 2.2.8. Water Vapor Permeability (WVP)

A W3/060 water vapor permeability tester was used, with 4 cycles and an interval of 60 min. During the test, the chitosan composite films were cut into circular pieces with a diameter of 8 cm, and the thickness of the film was measured with a micrometer. Then, it was placed in a moisture permeable cup, with a temperature of 25 °C and a humidity of 50% RH for testing. The WVP was calculated using Equation (3):

WVP(g·cm/cm^2^·s·Pa) = (Δm·X)/(S·ΔP·t) (3)

where Δm is the weight change of cup (g); X is the thickness of the film (cm); S is the effective area of the film (cm^2^); ΔP is the difference in partial water vapor pressure between the two sides of films (Pa); t is the time of the weight change (s).

#### 2.2.9. Water Contact Angle (WCA)

The WCA of the film surface was measured using a JC2000C1 contact angle measuring instrument at 25 °C. Three different positions of each film were measured; the volume of the water drop was 5 μL and each sample was measured three times.

#### 2.2.10. Statistical Analysis

Statistical analysis was carried out using Origin v2021 software (Origin Lab Corporation, USA) and SPSS (v17.0, Chicago, IL, USA) software. All statistical data were determined with one-way analysis of variance (ANOVA) and Duncan’s multiple tests using SPSS. Significant differences were defined as *p* ≤ 0.05.

## 3. Results and Discussion

### 3.1. Microstructural Morphology of Chitosan-Based Composite Films

#### 3.1.1. Fourier Transform Infrared (FT-IR) Spectroscopy

**Figure 2:**
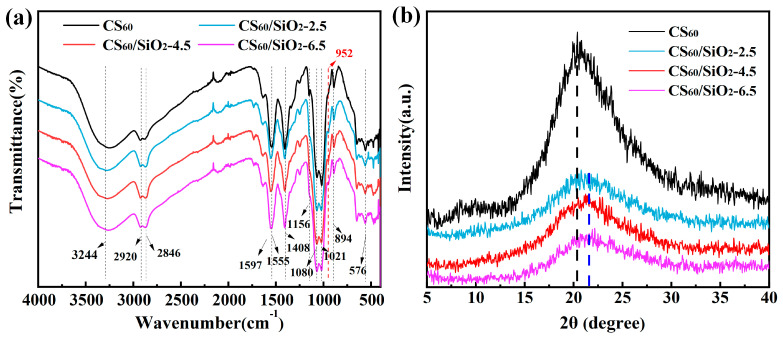
(**a**) FTIR and (**b**) XRD spectra of CS_60_ and chitosan/sorbitol/SiO_2_ composite films.

To investigate the structural differences between the chitosan-based composite films with and without SiO_2_, IR spectra were recorded. The FTIR spectra of CS_60_ and CS/sorbitol/SiO_2_ composite films are shown in Figure 2a. From the FTIR spectra curve of the CS_60_ composite film, it can be seen that 3244 cm^−1^ is the hydroxyl (O-H) stretching vibration absorption peak, and the peaks at 2921 cm^−1^ and 2882 cm^−1^ correspond to the two C-H stretching vibration absorption peaks. The absorption peak of the amide II band (N-H bending vibration) is found at 1597 cm^−1^; 1408 cm^−1^ corresponds to the bending and deformation absorption peaks of =CH_2_ and -CH_3_; 1156 cm^−1^ is the C-O stretching vibration absorption peak of the β-glucoside bond; 1080 cm^−1^ and 1021 cm^−1^ are the two stretching vibration absorption peaks of C-O. In the fingerprint area, 894 cm^−1^ corresponds to the absorption peak of C-O stretching vibration [30,31].

From the FTIR spectra curves of CS/sorbitol/SiO_2_ composite films in Figure 2a, the peak value between 3600 and 3000 cm^−1^ was caused by the stretching vibration of the amino (N-H) and hydroxyl (-OH) groups, widening the peak value of 3244 cm^−1^, indicating that nano-SiO_2_ enhanced the hydrogen bond between chitosan and sorbitol and improved their compatibility [12]. In addition, the positions of absorption peaks at 1597 cm^−1^, 1156 cm^−1^ and 894 cm^−1^ were unchanged, indicating that amino and β-glucoside bonds were not involved in the reaction. The absorption peak at 1095 cm^−1^ (C-O stretching vibration on C_3_) moved toward the low wave number of 1080 cm^−1^, and the absorption peak at the 1027 cm^−1^ wavelength (C-O stretching vibration on C_6_) moved in the low wave direction to 1021 cm^−1^ [32,33]. The movements of these two absorption peaks indicate that there was an intermolecular hydrogen bond between nano-SiO_2_ and chitosan molecules. Moreover, an absorption peak appeared at 952 cm^−1^, which might be the stretching vibration absorption peak of Si-OH [34]. It was indicated that nano-SiO_2_ grows on a molecular chain skeleton of the CS_60_ composites [23]. The functionalized nano-SiO_2_ could be dispersed in the CS_60_ composite film, which further improves the compatibility of chitosan and sorbitol.

#### 3.1.2. XRD Spectra Analysis

Figure 2b shows the XRD spectra of CS_60_ and CS/sorbitol/SiO_2_ composite films. In the CS_60_ film at 2θ = 10° and 2θ = 20°, there is a wide diffraction peak, which is a typical fingerprint of the chitosan/sorbitol composite film [35]. Additionally, the diffraction peak located at 2θ = 10° is recognized as the crystalline hydrate of chitosan, and the diffraction peak that appeared at 2θ = 20° is assigned to the anhydrous crystallization of chitosan. Consequently, the hydrogen bands between the molecular chains of chitosan affect the crystallization behavior of chitosan, and the molecular conformation of chitosan has a significant effect on the crystalline form of chitosan [36,37].

In the XRD spectra of CS/sorbitol/SiO_2_ composite films, the diffraction peaks of films at 10° all disappeared after nano-SiO_2_ was added. As the silicon dioxide content increased, the diffraction peak intensity at 2θ = 20° significantly decreased and gradually moved towards 2θ = 21.7°, indicating the less crystalline structure formed in the chitosan matrix. Nano-SiO_2_ that undergoes surface treatment contains numerous -Si-OH groups, forming strong hydrogen bonds with chitosan molecules, which influence molecular inter-chain interactions and the movement of chitosan to a certain extent, hindering the chitosan crystallization process. Song et al. [38] and Qiao et al. [39] both reported the disappearance of the peak at 10° and ascribed this to the strong hydrogen bonds between new additives and chitosan; the former pointed out that this destroyed the formation of hydrogen bonds between chitosan chains, while the latter explained that it limited the movement of chitosan chains. The synergistic effect of physically modified nanoSiO_2_ and -OH in sorbitol increases the free amino group in chitosan, which enables the double helix structure of the polysaccharide asymmetric unit of chitosan to become more unstable. The formation of an unstable architecture offers plentiful growth sites for the formation of amorphous nano-SiO_2_ [36,40]. Based on the results of FIIR and XRD, it was determined that the inorganic phase in the prepared CS/sorbitol/SiO_2_ films is amorphous silica [41].

#### 3.1.3. SEM of Chitosan-Based Composite Films

**Figure 3:**
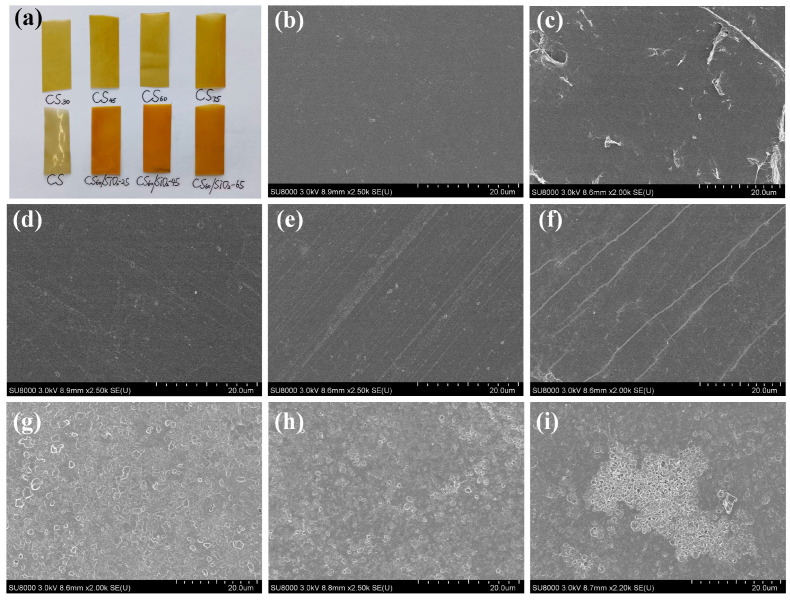
(**a**) Optical image of chitosan-based composite films. SEM photomicrographs of (**b**) CS, (**c**) CS_30_, (**d**) CS_45_, (**e**) CS_60_, (**f**) CS_75_, (**g**) CS_60_/SiO_2_-2.5, (**h**) CS_60_/SiO_2_-4.5; (**i**) CS_60_/SiO_2_-6.5.

Figure 3a shows an optical image of chitosan-based composite films. Among all films, CS has relatively high transparency and obvious folds on the surface. After adding sorbitol, the color deepens and the surface smoothness increases. The chitosan composite film improved by functionalized nano-silica is more flexible and elastic. SEM micrographs of the surface CS and CS/sorbitol composite films are presented in Figure 3b–f. The surface of pure chitosan film is smooth, continuous and dense. Here, the addition of sorbitol did not introduce discontinuities or porous structures into the film. The films are continuous and dense, which indicates that sorbitol has good miscibility and compatibility in chitosan [2]. When adding 30% sorbitol, the microsurface of the composite film is uneven, with many wrinkles and pores appearing. This implies that the combination of chitosan and sorbitol needs to be further improved, as only a small number of hydrogen bonds are formed with sorbitol due to the inadequate hydroxyl groups [42]. The surfaces of the CS_45_, CS_60_ and CS_75_ are relatively smooth compared with CS_30_, without obvious gaps and pores, which indicates that chitosan and sorbitol have good intermolecular compatibility and are tightly bound. This morphology is similar to that described by previous studies [43,44]. Moreover, sorbitol molecules effectively fill the network structure between chitosan molecules, forming a continuous and uniform film, and make the structure of chitosan matrix more orderly [15,45]. Figure 3g–i show the surface morphology of the CS/sorbitol/SiO_2_ composite films. Figure 3g shows that the surface of CS_60_/SiO_2_-2.5 is rough, with significant wrinkles and overall discontinuity. The addition of 45 mg nanoSiO_2_ is more well-distributed in the chitosan composite film due to the presence of a large number of hydrogen bonds between nanoSiO_2_ and the chitosan plasticizer system [46], resulting in the cross-linking network structure that formed between nanoSiO_2_ and the chitosan polymer matrix [47]. The CS_60_/SiO_2_-6.5 composite film’s surface is uneven and there are no obvious pores, but agglomeration occurs (Figure 3i). This is because the nano-SiO_2_ content is high, and in addition to forming intermolecular hydrogen bonds with chitosan and sorbitol, there are also unbound silicon hydroxyl groups that have a large surface energy and are prone to self-aggregation [48]. SiO_2_ is tightly deposited on the surface of chitosan, forming a non-uniform layer of silica nanoparticles [36]. A similar observation was reported by Hosseini et al. [49], who investigated the fish gelatin–chitosan nanoparticles composite and Chang et al. [50], who worked with starch–chitosan nanoparticles composites.

### 3.2. Mechanical Properties of Chitosan-Based Composite Films

**Figure 4:**
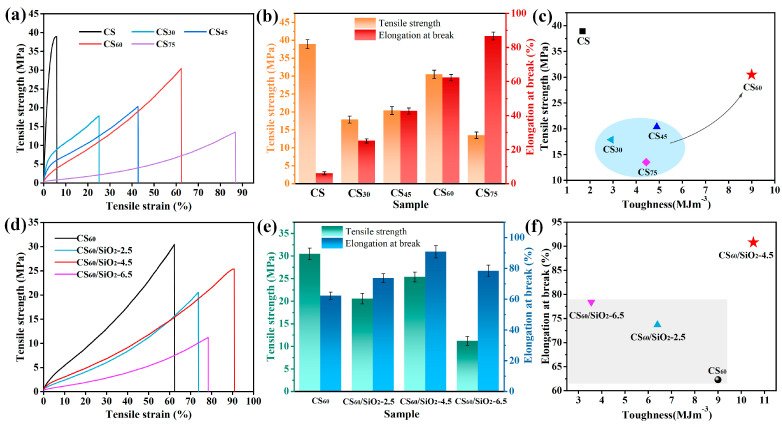
(**a**) Stress–strain curves, (**b**) bar chart of tensile strength and elongation at break, (**c**) toughness–tensile strength scatter plot of chitosan/sorbitol composite films. (**d**) Stress–strain curves, (**e**) bar chart of tensile strength and elongation at break, (**f**) toughness–elongation at break scatter plot of chitosan/SiO_2_ composite films.

Mechanical performance is an important factor in evaluating whether a film can be used for packaging, as it represents the durability and physical integrity of the film. The stressstrain curve of CS/sorbitol composite films is shown in Figure 4a. Figure 4b shows the corresponding bar charts of tensile strength and elongation at break. The tensile strength of CS film can be up to 38.91 MPa, which is the highest mechanical strength of all films, but the fracture elongation is only 5.9%, far from meeting the actual application requirements, and the toughness needs to be improved. The addition of sorbitol improved the mechanical properties of the chitosan composite film. Tensile strength, break elongation and toughness data for different chitosan-based films are shown in Table 1. The results show that with the gradual increase in sorbitol (≤75 wt %), the elongation at break of CS/sorbitol films significantly increased. The elongation at break of the CS/sorbitol composite film was 13 times higher than that of the pure CS film when the addition of sorbitol was 75 wt %.

In addition, the tensile strength and elongation at break of CS/sorbitol composite films gradually achieve a balance with the increase in sorbitol content, and toughness reaches the highest value when chitosan–sorbitol = 1:0.6 in this research. The low viscosity of plasticizers increases the flowability of biopolymer chains, leading to film structure softening and reducing tensile strength [51]. By adding sorbitol to the chitosan film, the hydrogen bonds formed between sorbitol molecules and chitosan will interact with the intermolecular and intramolecular hydrogen bonds formed by destroying the amino and hydroxyl groups of chitosan, reducing the intermolecular force of chitosan and enhancing the mobility of molecular chains in the film (with easier intermolecular relative slip), thereby increasing the flexibility of the composite films [43,48]. When the content of sorbitol reaches 75% that of chitosan, there is still good compatibility between sorbitol and chitosan [52]. The formation of a dense membrane further increased the spatial distance of the CS molecular chains, weakened the interchain force, increased the interchain free volume and reduced the tensile strength. However, the formation of a structurally dense film with a fracture elongation about 10 times that of the pure chitosan film fully reflects the plasticizing effect of hydroxyl (-OH) in polyol plasticizers. Therefore, the stress–strain curve exhibited the characteristics of a plastic deformation stage. An excessive dose of plasticizer can cause the plasticizer to aggregate and form small droplets, which are distributed in the network structure during the film-forming process. A stress concentration is easily formed when stretched, resulting in a serious decrease in tensile strength.

Figure 4c shows the scatter plot between the toughness and tensile strength of each film. The toughness of composite films can be calculated by the area under the tensile stress–strain curve [4]. The toughness of the pure CS film is only 1.67 ± 0.13 MJm^−3^. It is worth noting that the film toughness obviously increased with the addition of sorbitol: CS_30_ (2.91 ± 0.21 MJm^−3^), CS_45_ (4.89 ± 0.26 MJm^−3^), CS_75_ (4.43 ± 0.34 MJm^−3^), while the toughness of the CS_60_ film can reach 8.99 ± 0.4 MJm^−3^, which is more than five times that of the CS film. The toughness of the film significantly improved; the toughness was more thanfive times that of the pure chitosan film and superior to other films. A balance between a certain tensile strength and ideal elongation at break was also obtained. CS_60_ has ideal tensile strength, elongation at break, and toughness, and its comprehensive mechanical properties are the best.

By adding nanoparticles to change the aggregation morphology of chitosan molecules, the mechanical and barrier properties of chitosan materials can be further improved. The stress–strain curve of CS/sorbitol/SiO_2_ composite films is shown in Figure 4d. Figure 4e shows the corresponding bar charts of tensile strength and elongation at break. The data show that the addition of nano-SiO_2_ has a significant impact on the elongation at break of chitosan composite films. When the dosage of nano-SiO_2_ is less than 4.5%, the elongation at break of the composite film shows an increasing trend with the increase in dosage. When the amount of nano-SiO_2_ that is added is 4.5 wt %, its tensile strength is 24.5 MPa. The elongation at break of CS_60_/SiO_2_-45 can reach up to 90.8%, which is the largest of all films and 15 times higher than that of pure chitosan films. As a surface modifier of nano-silica, Tween-80 causes the hydrogen bond force between chitosan molecules to weaken in the presence of a plasticizer [53,54], so the tensile strength of CS_60_/SiO_2_ composite films is lower than that of CS_60_. Hou et al. [55] also improved the EAB of agar/sodium alginate films with the addition of 2.5–10% of nano-SiO_2_; they ascribed this enhancement to the strong interactions between nano-SiO_2_ and the matrix via hydrogen bonding. Nano-SiO_2_ with a large surface area tends to interact more with the hydroxyl groups and carboxylic groups of chitosan, which facilitated the transfer of stress from the matrix to the reinforcing phase via the interface. Wu et al. [56] improved the EAB of starch-based films with A_g_ nanoparticles through the van der Waals interactions between hydroxyl groups of starch and A_g_ nanoparticles. When the amount of SiO_2_ exceeds 4.5%, the elongation at break decreases. The reason for this is that as the amount of nano-SiO_2_ increases, SiO_2_ particles gradually aggregate and unevenly disperse, and agglomeration occurs, which disrupts the density of the film [57,58], causing defects such as “pores” to form in the composite film. During the stretching process, the sample first breaks at the “pores”. The introduction of nano-SiO_2_ reduces the tensile strength, but the elongation at break is greatly improved, improving the toughness and processability of the composite film.

Figure 4f shows that the toughness of CS_60_/SiO_2_-4.5 (10.52 ± 0.41 MJm^−3^) is significantly better than that of the other composite films: CS_60_/SiO_2_-2.5 (6.39 ± 0.28 MJm^−3^) and CS_60_/SiO_2_-6.5 (3.55 ± 0.19 MJm^−3^). In particular, the toughness of CS_60_/SiO_2_-4.5 is six times higher than that of pure CS film, which is a 17% improvement compared to CS_60_. The addition of nano-silica can not only improve the compatibility of chitosan and sorbitol and improve the elongation at break of the chitosan composite film, but also further improves the film-forming performance and toughness of the chitosan composite film.

### 3.3. Thermal Property

#### 3.3.1. Thermogravimetric Analysis

**Figure 5:**
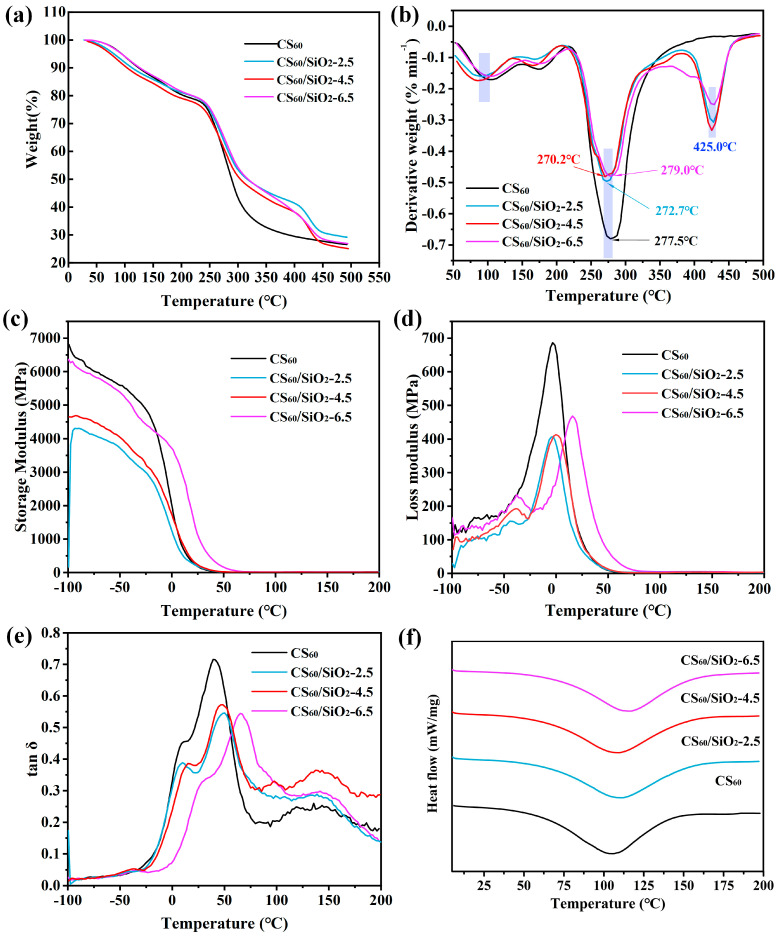
(**a**) TGA, (**b**) DTGA, (**c**) storage modulus, (**d**) loss modulus, (**e**) tan δ, (**f**) DSC curves of chitosan-based composite films.

Thermogravimetric analysis was used to study the weight variation of polymer films with increasing temperature. Figure 5a shows the thermogravimetric curves of CS_60_ and CS/sorbitol/SiO_2_ composite films. CS_60_ without the addition of nano-silica only has two weight loss stages, while the CS/sorbitol/SiO_2_ composite films present three weight loss stages. Stage 1: Between 30 and 200 °C, all curves show significant weight loss (about 20%), which is caused by the evaporation of free and bound water contained in the sample [30,59]. Stage 2: The significant weight loss of the CS_60_ film at from 200 °C to 350 °C is related to complex processes such as the thermal decomposition of chitosan and sorbitol [60,61]. For CS/sorbitol/SiO_2_ composite films, the weight loss in the temperature range of from 200 °C to 350 °C is significantly lower than that of the CS_60_ film, indicating that interface interactions such as the hydrogen bonds formed between nano-SiO_2_ and chitosan and sorbitol improved the thermal stability of chitosan-based composite films [62]. Furthermore, the DTG curves in Figure 5b suggest that all thermograms of CS/sorbitol/SiO_2_ composite films exhibited similar decomposition and weight loss patterns, with a maximum degradation rate around at 275 °C, but they were significantly lower than the degradation rate of CS_60_. Compared to CS_60_, CS/sorbitol/SiO_2_ composite films exhibit one more degradation stage in the range of 400–470 °C. The third stage: after 350 °C, the weight of CS/sorbitol/SiO_2_ composite films continued to slowly decrease, which can be attributed to the decomposition of C-C, C-O, and C-N single bonds in chitosan, as well as the thermal degradation of Tween-80 in modified nano-SiO_2_ micropores [36,63]. Nano-SiO_2_ can be considered to grow on the molecular chain skeleton of the CS_60_ composites, improving their thermal stability.

#### 3.3.2. Dynamic Mechanical Analysis and Differential Scanning Calorimetry

DMA is an effective tool for studying the relationship between polymer molecular chains, structure, and thermal–mechanical performance. Figure 5c–e depict the dynamic thermal behavior of the samples, including storage modulus, loss modulus and tan δ. The storage modulus decreased with the addition of SiO_2_ 25, 45 mg, and increased with the addition of 65 mg. This suggests that the addition of SiO_2_ may improve the storage modulus of certain content. The two peaks in Tan δ curves correspond to two glass transition temperatures (T_g_); the first strong peak of CS_60_ appeared at 30 °C, while the second small peak of CS_60_ appeared at 126 °C. The addition of nano-SiO_2_ improved the T_g_ of the film; the largest increase was observed in CS_60_/SiO_2_-6.5, where the first peak shifted to 62 °C. Qiao et al. [64] obtained similar results for two T_g_ of chitosan films solved in three acid solutions at around 55 °C and 125 °C. Ma et al. [45] also observed two T_g_ in a chitosan/sorbitol film, which appeared at around 15 °C and 103 °C. Liu et al. [60] reported two peaks in tan δ curves of chitosan film using DMA; he defined the first peak as β-relaxation related to the motions of the side chains, while only the second peak was associated with the T_g_ of chitosan films. The different chitin source, extraction methods, molecular weights, viscosities, degrees of deacetylation and film-forming methods resulted in the divergent results in measurements of T_g_ in chitosan film. Previous studies reported several single-T_g_ chitosan films including, 54–86 °C [65], 103 °C [39], 160 °C [66]. Table 2 shows the glass transition temperature of each film based on the DMA results.

A single endothermic peak in the range of 75–150 °C can be seen in DSC curves, as shown in Figure 5f, corresponding to non-freezing water locked in the amorphous region of the chitosan matrix [67]. Both amino and carboxyl groups can easily bind to water molecules in chitosan, and the water bound by the amino group evaporated faster than that held by carboxyl groups [65]. After the addition of hydrophilic nano-SiO_2_ containing a large number of hydroxyl groups, the water locked in the matrix evaporated more slowly, which explains the increase in the endothermic peak.

### 3.4. Water Vapor Permeability

**Figure 6:**
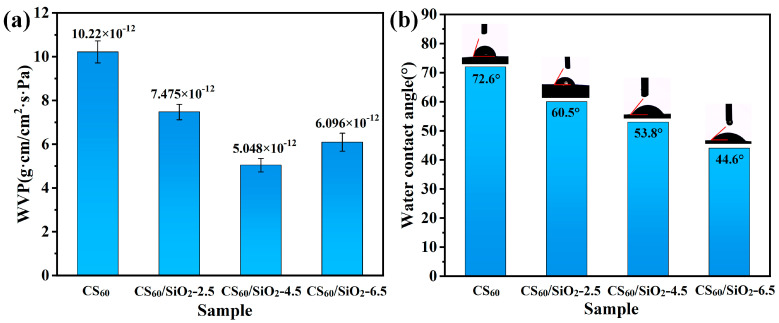
(**a**) WVP and (**b**) WCA of chitosan-based composite films.

The WVP of the film determines the water transfer between the surrounding atmosphere and the packaging environment. The permeability of a thin film largely depends on its chemical structure, morphology, and hydrophilicity. Figure 6a shows the water vapor transmittance of CS_60_ and CS/sorbitol/SiO_2_ composite films. The WVP of a CS_60_ composite film (10.22 × 10^−12^ g·cm/cm^2^·s·Pa) without the addition of nano-SiO_2_ is significantly higher than CS_60_/SiO_2_-2.5 (7.475 × 10^−12^ g·cm/cm^2^·s·Pa), CS_60_/SiO_2_-4.5 (5.048 × 10^−12^ g·cm/cm^2^·s·Pa) and CS_60_/SiO_2_-6.5 (6.096 × 10^−12^ g·cm/cm^2^·s·Pa). The overall trend and magnitude of WVP in the film samples are similar to the results of Dong et al. [48,68,69]. The composite film with added nanoparticles has a significantly higher moisture resistance than CS_60_. With the addition of nano-SiO_2_ (when the amount of nano-SiO_2_ is less than 6.5 wt %), the WVP of CS/sorbitol/SiO_2_ composite films gradually decreases, and a dense network structure is formed by secondary bonds, possibly due to the good dispersion of nano-SiO_2_ within chitosan molecules and good compatibility of each component [48]. In addition, due to the hydrogen bonding between nano-SiO_2_ and CS, as well as the uniformly distributed impermeable nano-particle layer, water vapor is forced to pass through the film through a more tortuous path, thereby increasing the effective path length for the diffusion and improvements in the WVP of the chitosan composite film [57,60,70]. Adding 6.5 wt % of nano-SiO_2_, an excessive amount, results in a large number of hydroxyl groups forming on the surface of nano-SiO_2_, which has high surface energy and is prone to uneven dispersion and aggregation (as shown in Figure 3i), resulting in a discontinuous structure with chitosan molecules. Water molecules are more likely to pass through, resulting in an increase in the moisture permeability coefficient.

### 3.5. Water Contact Angle

The surface water contact angle is an important parameter reflecting the wetting characteristics of film materials. When a droplet forms on a solid surface, wetting occurs. The droplet first contacts the substrate, forming a solid–liquid–gas three-phase contact line. Then, the three-phase contact line continues to move forward and is stable or metastable at a certain radius. The WCA values of chitosan composite films without nano-SiO_2_ and with different amounts of nano-SiO_2_ are shown in Figure 6b. The WCA value of the CS_60_ film is 72.6 ± 0.83°, which is higher than that of the chitosan composite film containing nano-SiO_2_: CS_60_/SiO_2_-2.5 (60.5 ± 0.54°), CS_60_/SiO_2_-4.5 (53.8 ± 0.75°), CS_60_/SiO_2_-6.5 (44.6 ± 0.69°).

The presence of silica particles reduces the WCA of the film, indicating that the addition of SiO_2_ reduces the surface hydrophobicity of the chitosan composite film. This is due to the large number of hydroxyl groups and high hydrophilicity of SiO_2_ nanoparticles on the surface of the composite film, which occupy a considerable area (as shown in Figure 3). When water droplets come into contact with the film, they quickly diffuse due to the attraction of hydroxyl groups [57,71]. Martínez-Aguilar et al. [72] added nano-SiO_2_ to a PLA film, observing that the WCA of the film decreased. Yu et al. [71] constructed a superhydrophilic surface with nano-SiO_2_ on chitosan film; the WCA of the film decreased from 66.1° to 3°. They both emphasized the importance of hydroxyl groups in nano-SiO_2_.

## 4. Conclusions

The chitosan-based nanocomposite films were prepared with sorbitol as a plasticizer and nano-SiO_2_ as the reinforcing agent via the solvent casting method, assisted by ultrasonication. The sorbitol showed a good plasticizing effect on pure chitosan film while the elongation at break of CS_75_ increased from 5.9% to >80%. The CS_60_ with the best toughness was selected as the subsequent nanocomposite’s matrix. When mixed with nano-SiO_2,_ the WVP and thermal stability of the chitosan film both improved while the WCA of the film decreased, turning the film into hydrophilic material. Furthermore, strong hydrogen bonds between nano-SiO_2_ and chitosan could reduce the crystallinity of the film. The CS_60_/SiO_2_-4.5 presented excellent physicochemical properties, with the largest elongation at break of 90.8% (14 times that of pure chitosan) and toughness of 10.52 MJm^−3^ (6 times that of pure chitosan). This paper has good practical guiding significance for broadening the application of chitosan composite films and the industrial production of biomass plastics.

## Figures and Tables

**Table 1:** Tensile strength, break elongation and toughness data of different chitosan-based films. Pure chitosan (CS), chitosan with 30% (*w*/*w*) sorbitol (CS_30_), 45% (*w*/*w*) sorbitol (CS_45_), 60% (*w*/*w*) sorbitol (CS_60_), 75% (*w*/*w*) sorbitol (CS_75_). Chitosan with 0.60 g sorbitol and 2.5% (*w*/*w*) nano-SiO_2_ (CS/SiO_2_-2.5), 4.5% (*w*/*w*) nano-SiO_2_ (CS/SiO_2_-4.5), 6.5% (*w*/*w*) nano-SiO_2_ (CS/SiO_2_-6.5), respectively.

Sample	CS	CS_30_	CS_45_	CS_60_	CS_75_	CS_60_/SiO_2_-2.5	CS_60_/SiO_2_-4.5	CS_60_/SiO_2_-6.5
TS/MPa	38.91	15.68	19.11	29.27	13.22	20.56	25.38	11.22
EAB/%	5.9	20.96	42.68	66.16	87.23	73.7	90.8	78.4
Toughness/MJm^−3^	1.67	2.91	4.89	8.99	4.43	6.39	10.52	3.55

**Table 2:** The glass transition temperature (T_g_) of samples obtained using DMA.

Sample	T_g1_ (°C)	T_g2_ (°C)
CS_60_	30	123
CS_60_/SiO_2_-2.5	50	129
CS6_0_/SiO_2_-4.5	52	132
CS_60_/SiO_2_-6.5	62	135

## Data Availability

The authors do not have permission to share data.
